# Implications of SNP-triggered miRNA dysregulation in Schizophrenia development

**DOI:** 10.3389/fgene.2024.1321232

**Published:** 2024-01-26

**Authors:** Fadumo Abdullahi Mohamed, Kristine Freude

**Affiliations:** Department of Veterinary and Animal Sciences, Faculty of Health and Medical Sciences, University of Copenhagen, Frederiksberg, Denmark

**Keywords:** Schizophrenia, microRNA, SNP, dopamine, glutamate, GABA

## Abstract

This review examines the substantial involvement of Single Nucleotide Polymorphisms (SNPs) and microRNAs (miRNAs) in the etiology and susceptibility to Schizophrenia, with particular emphasis on the dopaminergic, glutamatergic, and GABAergic systems. It elucidates the potential of SNPs to disrupt miRNA-mRNA interactions, leading to alterations in the regulatory mechanisms of Schizophrenia risk genes and subsequently influencing the susceptibility to Schizophrenia. Specific attention is given to the impact of SNPs in *DICER, DROSHA*, and *DGCR8*, as well as the potential for changes in *DRD2* gene expression driven by miR-9 and miR-326, heightening the likelihood of Schizophrenia development. Furthermore, the review explores genetic alterations in the glutamatergic system, focusing on modifications linked to *GRIN2A* and its associated miRNAs, which have been reported to have a notable impact on the occurrence of Schizophrenia. Knowledge of the involvement of SNPs within miRNAs in influencing the expression of essential genes within the GABA system are emerging and described in this review, including their potential consequences for Schizophrenia.

## Graphical Abstract

**Figure:**
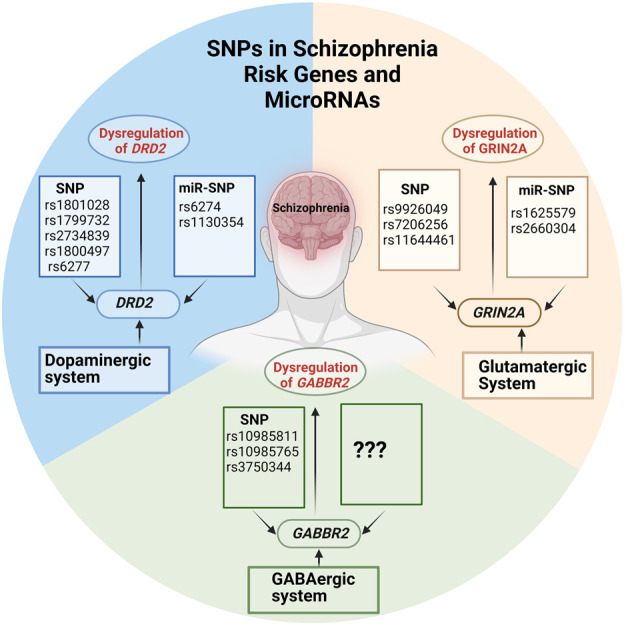
Created with BioRender.com.

## 1 Introduction

Schizophrenia (SCZ) is a profound mental disorder that impacts around 1% of the global population. Despite its relatively low prevalence, this condition exerts a substantial burden on affected individuals, their families, healthcare systems, and society. People living with SCZ typically face a shortened life expectancy by 15–20 years than that of the general population (Stępnicki et al., 2018; Peritogiannis et al., 2022).

This shortend lifespan is primarily correlated with elevated suicide rates and suicide attempts. It has become increasingly evident that this heightened risk of suicidal tendencies is closely connected to concurrent conditions, including major depression (MD) and substance use disorders (SUDs) (Onaemo et al., 2022). Considering the extensive array of genes linked to SCZ, along with comorbidities such as MD and SUDs, as well as environmental factors, it becomes evident that risk genes play a significant role. However, it is essential to emphasize that other epigenetic factors play an equally crucial role in the onset and progression of SCZ, including the altered expression of genes associated with the machinery responsible for microRNA biogenesis (Rey et al., 2020). Until now, the exact cause of SCZ remains elusive, despite its widespread occurrence and significant impact on the healthcare sector. Over the past few decades, research has largely concentrated on postmortem brain analysis and neuroimaging, reinforcing the idea that SCZ stems from the formation of irregular neuronal connectivity and signal processing occurring in early adulthood. This has led to its interpretation as a neurodegenerative disorder (Csernansky, 2007). Nevertheless, our comprehension of SCZ has recently undergone a substantial transformation. It has shifted away from perceiving it as a degenerative disorder emerging in early adulthood, to acknowledging it as a neurodevelopmental condition with origins predating birth (De Berardis et al., 2021). The neurodevelopmental theory finds support in genetic and environmental investigations conducted on families and twins. These studies suggest that both environmental and genetic risk factors exert their influence during prenatal, perinatal, and early adolescent phases. This impact alters the developmental course, ultimately culminating in the manifestation of SCZ during adolescence and early adulthood (Birnbaum and Weinberger, 2017; Trifu et al., 2020). Additionally, the neurodevelopmental perspective on SCZ recognizes that the existence of SNPs in risk genes significantly raises the likelihood of SCZ development. To date, genome-wide association studies (GWAS) have identified over 180 loci strongly associated with SCZ (Schizophrenia Working Group of the Psychiatric Genomics Consortium, 2014; Singh et al., 2022; Trubetskoy et al., 2022). SNPs refer to variations in a single nucleotide within the DNA sequence, such as substituting Adenine (A) with Thymine (T), Cytosine (C), or Guanine(G). These genetic variations play a crucial role in individual diversity and can influence phenotypes, traits, and disease susceptibility (Schork et al., 2000). SNPs are frequently located within genomic regions subject to the influence of natural selection, which favors specific alleles conferring genetic adaptations. Interestingly, the majority of SNPs associated with human disease risk identified through GWAS do not occur in protein-coding regions but rather in regions that regulate gene transcription levels (Coetzee et al., 2012; Kumar et al., 2013; Gibbons et al., 2018). This implies that SNPs located in non-coding regions responsible for regulatory functions might exhibit a robust link to diseases such as SCZ (Gibbons et al., 2018). Epigenetic factors without coding functions where SNPs have been identified include microRNAs (miRNAs). miRNAs are complementary short segments of the protein coding mRNAs and can act as transcriptional repressors through destabilizing the mRNA and thereby reducing the amount of transcript of a given gene to be translated into protein. Numerous studies have provided evidence of miRNAs playing a role in the development of SCZ (Beveridge et al., 2008; Shorter and Miller, 2015; Hu et al., 2019; Zhao et al., 2019). Further support of the significant role of miRNAs in SCZ is derived from the discovery that SCZ-risk genes exhibit a higher count of predicted miRNA-binding sites compared to unrelated protein-coding genes (Hauberg et al., 2016). As previously mentioned, environmental factors can further contribute to the onset of SCZ. For instance, exposure to substances involved in substance use disorders (such as alcohol and drugs) has been associated with changes in miRNA levels in the brain, potentially elevating the risk of SCZ development (Thomas and Zakharenko, 2021).

## 2 Genetic complexity of SCZ

The intricate genetic nature of SCZ is underscored by meta-analysis studies involving twins and families, revealing an 80% heritability rate, confirming that genetics is the primary risk factor for developing SCZ. However, the absence of a clearly defined mode of inheritance is evident, as fewer than one-third of SCZ patients have a family history of the condition. This ambiguity complicates the task of elucidating the origins of SCZ (Tiwari et al., 2010; Chou et al., 2017; Cheng et al., 2021). Nonetheless, with the recognition of SCZ having a polygenic inheritance pattern and the presence of polymorphic markers for genetic mapping, numerous endeavors have been made to identify genes that confer susceptibility to the disorder through linkage or association methods (Yan et al., 2016; Greenwood et al., 2019). However, the most efficient approach to uncovering genetic risk factors for SCZ continues to pose a challenge, given that each new study brings to light additional SNPs within different genes associated with a heightened risk of developing SCZ. To date, numerous genes associated with the risk of SCZ have been identified, and over 100 genetic loci have been examined for their potential links to these SCZ risk genes (Schizophrenia Working Group of the Psychiatric Genomics Consortium*, 2014; Singh et al., 2022; Trubetskoy et al., 2022).

The largest GWAS analysis has used data from 69369 subjects with SCZ, and 236642 controls and identified a total of 313 SNPs in 263 independent loci, which are reportedly associated SCZ. In this study, fine-mapping analyses were carried out, identifying a total of 628 genes, among which 435 were associated with protein coding genes, each harboring at least one SNP. They further revealed that genes important for neurodevelopment were dysregulated in SCZ (Trubetskoy et al., 2022). Some of the genes, such as *GRIN2A*, which was previously described to be dysregulated (Trubetskoy et al., 2022), were confirmed to be dysregulated in two independent large-scale GWAS studies (Schizophrenia Working Group of the Psychiatric Genomics Consortium*, 2014; Singh et al., 2022). The most recent meta-analyses study interrogated whole exomes from a large cohort of 24248 SCZ patients and 97322 unaffected control individuals. The study reported very rare coding variants in ten genes, associated with an increased risk for development of SCZ (Singh et al., 2022). Among the multitude of genes identified as increasing the susceptibility to SCZ are genes that hold a significant function in the dopaminergic, glutamatergic, and GABAergic neurotransmitter systems (Sigvard et al., 2023). SCZ is theorized to be linked to imbalances within these specific systems. A recent study conducted by Sigvard and colleagues emphasized the pivotal role of all three systems in SCZ development (Sigvard et al., 2023). The research revealed a dynamic interaction among these three systems, marked by variations in dopamine synthesis capacity, reduced GABA levels in the anterior cingulate cortex (ACC), and alterations in glutamate levels within the thalamus or ACC. Furthermore, they argue that the combination of these three essential biological markers has the capacity to predict a patient’s condition, such as psychosis, a task that individual neurotransmitters alone may not be able to achieve. This suggests that a diagnosis of SCZ is more likely related to disruption in interconnections within brain macrocircuits rather than isolated abnormalities of specific neurotransmitters (Gaskin et al., 2016). Among the risk genes identified in these studies, which have been shown to have multiple risk-associated SNPs, are *Dopamine receptor D2* (*DRD2*)*, gamma-aminobutyric acid type B receptor subunit 2* (*GABBR2*), *glutamate ionotropic receptor AMPA type subunit 3* (*GRIA3*) and *glutamate ionotropic receptor NMDA type subunit 2A* (*GRIN2A*). The presence of variants in these genes, as identified in large GWAS studies, reinforces the idea that there is an intricate interplay among the dopaminergic, glutamatergic, and GABAergic systems in the development of SCZ (Schizophrenia Working Group of the Psychiatric Genomics Consortium*, 2014; Singh et al., 2022; Trubetskoy et al., 2022; Sigvard et al., 2023).

### 2.1 SNPs in SCZ risk genes affecting the dopaminergic system

*DRD2* has been shown to be an important player in the development of SCZ according to several studies (Yao et al., 2015; Benjamin et al., 2022; Alfimova et al., 2023). *DRD2*, which codes for the type-2 dopamine receptor (D2R) is an important G-coupled presynaptic receptor of the dopaminergic system (Oda et al., 2015). Several SNPs in *DRD2* have been identified as promising risk SNPs associated with the development SCZ. Among the identified SNPs are rs1801028, rs1799732, rs2734839 rs1800497, and rs6277 (Fan et al., 2010; Yao et al., 2015). Therefore, SNPs in *DRD2* might alter the receptor subunit confirmation leading to either more or less sensitivity to dopamine in SCZ (He et al., 2016). One specific example is SNP rs1801028, which results in a C>G change in the *DRD2* mRNA (NM_00795.4) at position 311. This results in the substitution of serine with cysteine in the DRD2 protein. Additionally, *in vitro* experiments have demonstrated that the rs1799732 SNP, located in the promoter region of *DRD2*, influences gene expression (Yao et al., 2015). The rs2734839 variant within the *DRD2* gene is characterized by an A>G change and has been proposed to be significantly associated with SCZ. Furthermore, it has been established that this variant is not only significantly linked to SCZ, but also to a later onset of the disease (Voisey et al., 2012; Frydecka et al., 2021). The rs1800497 was initially believed to reside in the 3′untranslated region (UTR) of *DRD2*. However, it has been identified within the ankyrin repeat and kinase domain-containing 1 gene, which is situated approximately 10 kilobases downstream of *DRD2*. Even though the SNP is not located in the protein coding sequence, the proximity of this SNP to *DRD2* in the genomic region has been shown to lower the number of DRD2 receptor (Doehring et al., 2009; Li et al., 2018a). While the genetic makeup of the dopaminergic system plays a significant role in the pathophysiology of SCZ, the intricate connection between the dopaminergic system and the glutamatergic system has prompted further exploration of the role of the glutamatergic system in the development of SCZ (Konradi and Heckers, 2003).

### 2.2 SNPs in SCZ risk genes affecting the glutamatergic system

Recent GWAS studies have highlighted the significant involvement of SNPs located in *GRIA3* and *GRIN2A* in the functioning of the glutamatergic system (Schizophrenia Working Group of the Psychiatric Genomics Consortium*, 2014; Singh et al., 2022; Trubetskoy et al., 2022). *GRIA3* encodes for a subunit of the tetrameric AMPA-sensitive glutamate receptor. As a member of the glutamate receptor family, it plays a crucial role in depolarizing excitatory neurotransmission and facilitating synaptic plasticity within the mammalian brain (Magri et al., 2008; MacDonald et al., 2015). More specifically, *GRIA3* AMPA-receptor mutations have been associated with intellectual impairment and SCZ. Additionally, as glutamate receptors play a significant role in forming synapses, any abnormalities in these receptors might contribute to observed changes in decreased dendritic length, spine density and synaptic structures. These changes are commonly reported in many brain regions of patients with SCZ (Magri et al., 2008). SNPs in genes important for glutamate receptors might therefore contribute to the increased risk of SCZ. Another glutamate receptor implicated in the pathophysiology of SCZ is the GRIN2A. *GRIN2A* encodes the glutamate receptor subunit epsilon 1 protein associated with NMDA receptors. NMDA receptors are primarily expressed during childhood and adolescence (Poltavskaya et al., 2021). Working alongside AMPA receptors, NMDA receptors are instrumental in processes such as learning, memory, and synaptic plasticity, including long-term potentiation (Poltavskaya et al., 2021). Blockage of the AMPA and NMDA receptors in mature neural systems has been shown to decrease the density of dendritic spines which is then postulated to result in SCZ like symptoms and cognitive impairments (Hu et al., 2015; Poltavskaya et al., 2021) Furthermore, the published GWAS studies suggest that *GRIA3* and *GRIN2A* to be dysregulated. SNPs in both genes have been shown to confer a substantial risk to develop SCZ (Singh et al., 2022; Trubetskoy et al., 2022). In the GWAS study conducted by Singh et al., numerous common variants were identified, including a particularly rare variant for the AMPA receptor subunit *GRIA3*, which exhibited significant risk (*p* = 5.98 × 10^−7^), and the *GRIN2A* variant rs9926049, with a *p*-value of 1.57 × 10^−10^) (Singh et al., 2022). Furthermore, the variants rs7206256 and rs11644461 in *GRIN2A* have been shown to be involved in early onset SCZ in Russian and Serbian populations (Poltavskaya et al., 2021). In an American study that included subjects from diverse backgrounds, it was observed that these identical variants were notably more prevalent in the early onset psychosis group when compared to the control subjects (Hojlo et al., 2023). Although the studies describe variants in *GRIN2A* to increase SCZ risk they do not specify how these variants affect gene function and expression.

### 2.3 SNPs in SCZ risk gene affecting the GABA system

The *GABBR2* gene, a key player in the GABA system, has been associated with the underlying mechanisms of SCZ (Trubetskoy et al., 2022). The GABBR2 receptor exhibits a strong binding affinity for GABA and is situated on the exterior of synapses. This receptor, along with its counterpart GABBR1, plays a pivotal role in governing neuronal network activity, neurodevelopment, and synaptic plasticity throughout the entire brain, as it is broadly distributed in the brain. Notably, GABBR1 and GABBR2 receptors have been observed to be significantly diminished in several brain regions (including the cerebellum, hippocampus, and entorhinal cortex) in individuals diagnosed with SCZ (Xu and Wong, 2018). The SNP rs10985811 within the *GABBR2* receptor gene has been identified to elevate the risk of developing SCZ. On the other hand, the rs10985765 SNP, located in exon 18 is causing a missense variation in the cytoplasmic domain of the receptor and was found to be associated with SCZ risk. The rs3750344 SNP, a synonymous variant found in exon 2, is believed to interfere with the binding of agonists or antagonists to the receptor (Miyazawa et al., 2022; Trubetskoy et al., 2022). Both studies provide evidence that SNPs in *GABBR2* reduce the number of GABA receptors present in SCZ patients. However, further studies are necessary to confirm the link between the specific SNPs and reduced GABA receptor expression in SCZ.

While SNPs within genes are known to play a pivotal role in the risk of SCZ, it is worth noting that SNPs situated in the 3′untranslated region (3′UTR) of mRNA have a substantial impact on gene expression by influencing the interaction between miRNAs and their target sites on the mRNA. Specifically, a small segment of the miRNA sequence, known as the seed region and positioned at positions 2-7 from the 5′end of the miRNA, pairs with complementary sequences in the 3′UTR region of the mRNA. This 3′UTR region is crucial for recognizing and binding to miRNA targets. Consequently, SNPs located in the 3′UTR region have the potential to obstruct, weaken, or even create new miRNA binding sites (Bruno et al., 2012; Haas et al., 2012). Even though SNPs have the capacity to alter the binding sites of miRNAs, they have been found within 3′UTR region of SCZ risk genes, underscoring their significant role in the onset and progression of SCZ (He et al., 2021; Zhang et al., 2021) It is important to acknowledge that miRNAs, even in their non-mutated form, have a vital impact on gene expression (O’Brien et al., 2018).

## 3 Biogenesis and mechanism of miRNAs

miRNAs are short single stranded non-coding RNA molecules which consist of 18–22 nucleotides. miRNAs are dispersed throughout the human genome however 70% of identified miRNAs are only expressed in the brain indicating their importance for brain development (Chen and Qin, 2015). miRNAs function as gene regulators. A single miRNA can target the expression of large set of genes while expression of one gene can be regulated by several miRNAs (Zhang et al., 2023). As such, precise regulation of gene expression at various levels of neurodevelopment is performed by miRNAs. Previous research has identified miRNAs that are either neuron-specific or abundantly expressed in the brain, and they have been demonstrated to govern various facets of neuronal development. This encompasses the regulation of neural progenitor cell proliferation, determination of neuronal destiny, coordination of circuitry establishment, impact on synaptic plasticity, and the maintenance of overall brain health (Nguyen et al., 2018; Nowakowski et al., 2018; Wang et al., 2022). Therefore, it is imperative to maintain a well-functioning miRNA biogenesis process for the proper development of a healthy brain. Any disruption in the biogenesis of miRNAs has been associated with brain diseases, including SCZ (Beveridge et al., 2008; Shorter and Miller, 2015; Hu et al., 2019). The biogenesis of miRNA starts with the processing of RNA polymerase II/III transcripts post- or co-transcriptionally. Most well-studied miRNAs are derived mainly from intronic regions, with only a minor proportion originating from exons of protein-coding genes. In contrast, the rest of the miRNAs are intergenic, meaning they are generated independently of a host gene and are regulated by their own promoters. miRNA biogenesis is classified into two main categories: canonical and non-canonical biogenesis (O’Brien et al., 2018).

### 3.1 Canonical biogenesis pathway

**FIGURE 1:**
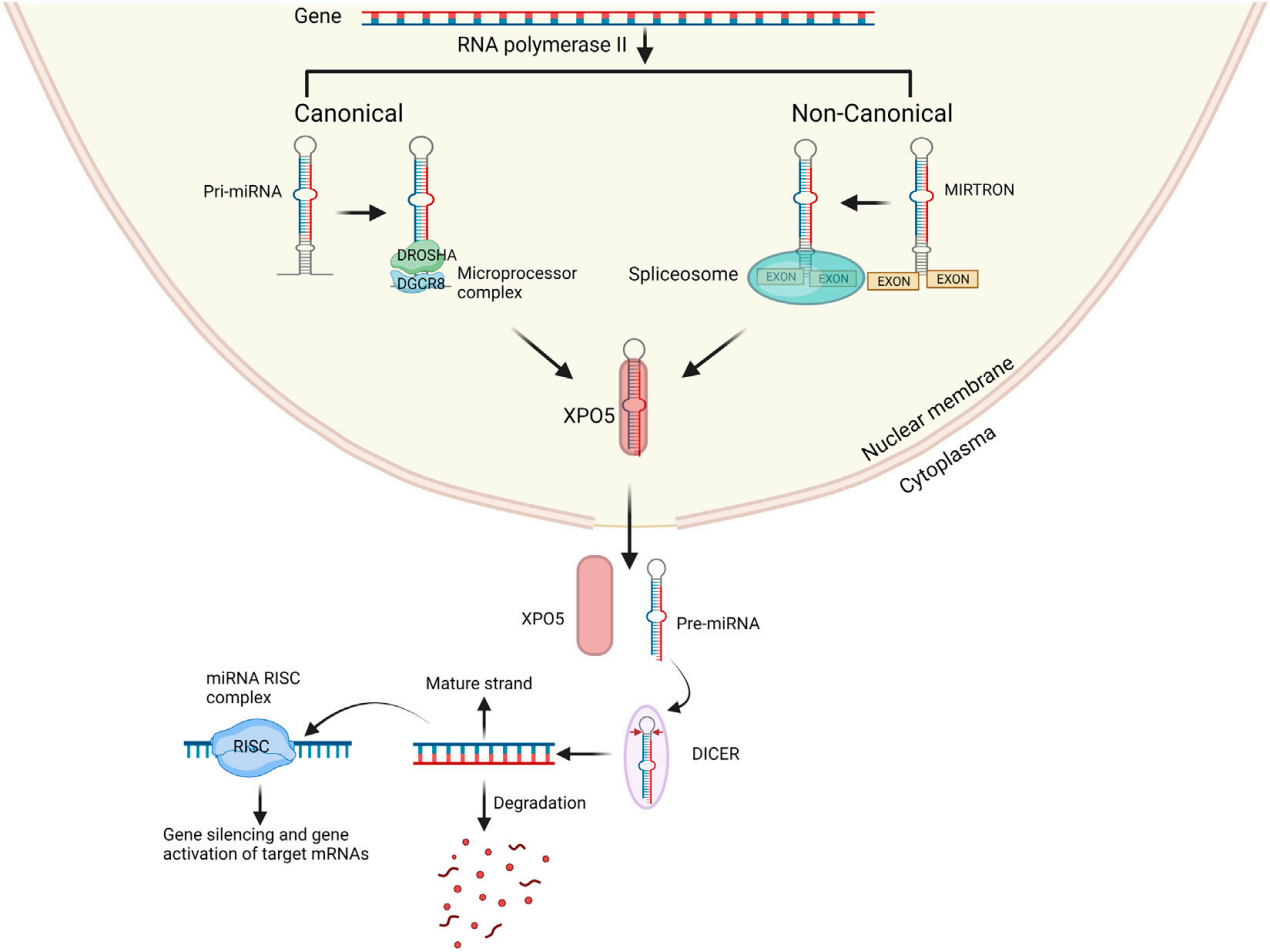
Overview of canonical and non-canonical miRNA biogenesis pathway. In the canonical pathway miRNA biogenesis begins with the production of pri-miRNA from their genes by RNA polymerases II. The microprocessor complex, which consists of DROSHA and DGCR8, cleaves the pri-miRNA to generate the pre-miRNA. In the non-canonical pathway mirtrons are processed from exons and then processed by spliceosomes into pre-miRNA. The pre-miRNA is transported to the cytoplasm by XPO5 and then processed by DICER into miRNA duplex. One strand either 3′ end or 5′ is incorporated into the RISC complex the remaining strand is degraded. Created with BioRender.com.

miRNAs are primarily processed in the canonical biogenesis pathway. Pri-miRNAs are transcribed from their genes by RNA polymerase III. The pri-miRNA is then further processed by the microprocessor complex consisting of RNase III domain-containing protein DROSHA and the RNA-binding protein encoded by the DiGeorge Syndrome Critical Region Gene 8 (DGCR8) into a ∼70-nt precursor miRNA (pre-miRNA). The pre-miRNA is subsequently transported to the cytoplasm by the nuclear export protein Exportin 5 (XPO5) (Figure 1). In the cytoplasm the pre-miRNA is further processed by the RNase III protein, DICER, which forms the mature 21-28 nucleotides long miRNA duplex (O’Brien et al., 2018; Suster and Feng, 2021). Both strands can individually act as gene regulator. The nomenclature of the miRNA strand is defined by the directionality of the mature miRNA strand. The 3p mature miRNA form is derived by the 3′ end of the pre-miRNA hairpin while the 5p strand is derived from the 5′end (O’Brien et al., 2018). The distribution of AGO-loaded 3p or 5p strands is contingent on the specific cell type or cellular environment, ranging from an even split to a significant preference for either one (O’Brien et al., 2018). The choice of which strand is incorporated into the RNA-induced silencing complex (RISC), composed of DICER, TAR RNA binding protein, and a member of the argonaute (AGO) family, is determined by the thermodynamic stability at the 5′end or the presence of a 5′Uracil nucleotide in the miRNA duplex. The strand with lower stability at the 5′end or a 5′Uracil is preferentially loaded into the RISC complex (Khvorova et al., 2003). The RISC complex with the mature miRNA controls the expression of specific genes by attaching to the 3′UTR region on the mRNA target through recognition of the complimentary ‘seed’ region of the mRNA. The miRNA-RISC complex uses nucleotide 2-8 in the miRNA to the seed match site in the target mRNA in the 3′UTR region. Although, many only state the ‘seed’ region to be in 3′UTR it has also been stated that some miRNAs control expression by specifically targeting the 5′UTR region, promotor region and/or coding region of some mRNAs (Rani and Sengar, 2022; Zhang et al., 2023).

### 3.2 Non-canonical biogenesis pathway

Several non-canonical miRNA biogenesis pathways have been identified. The non-canonical pathway uses various protein combinations from the canonical pathway, namely, DROSHA, DICER, XPO5, and AGO2. Non-canonical miRNA biogenesis can be divided into two categories: DROSHA/DGCR8-independent and DICER-independent mechanisms. An example of non-canonical miRNAs are mitrons. These mirtrons bypass the processing of DROSHA/DGCR8 and are instead processed by spliceosomes, allowing to produce functional miRNAs. Whilst bypassing of DICER creates non-functional miRNAs (Abdelfattah et al., 2014) the mirtons are exported into the cytoplasm by XPO5 and then processed by DICER to form mature miRNA after previous nuclear processing, including splicing (Figure 1) (Goymer, 2007; Havens et al., 2012; Rorbach et al., 2018). Although we have a clear understanding of the roles of canonical miRNAs, the functions of non-canonical miRNAs remain elusive, and we are still in the process of unraveling their involvement in cellular processes and disease pathogenesis (Abdelfattah et al., 2014). Traditionally, canonical miRNA function was primarily associated with gene silencing. However, it has come to light that miRNAs can also influence gene expression positively. Remarkably, a single miRNA can both silence and upregulate various genes (Valinezhad Orang et al., 2014).

### 3.3 miRNA-mediated gene silencing

As previously mentioned, miRNA’s gene silencing mechanism is mediated by the miRNA-RISC complex. miRNAs utilize their miRNA response elements (MREs) to bind to their target mRNAs. They guide the miRISC complex to complementary sequences, typically located in the 3′UTR of mRNAs. This seed match site allows a single miRNA to potentially target multiple mRNAs within the 3′UTR region, while, conversely, several miRNAs can target a single mRNA. When the miRISC complex binds to the target mRNA, it undergoes a conformational change, enabling extended seed pairing of the miRNA. This, in turn, results in more nucleotides interacting with the target mRNA, furthering the gene silencing process (Yan et al., 2018). Once the miRISC complex, typically composed of AGO proteins and GW182, is bound to the target mRNA, it can exert its influence on gene expression through mechanisms such as mRNA degradation, translational repression, or deadenylation (O’Brien et al., 2018). To facilitate mRNA degradation following the identification of the target mRNA, AGO proteins within the miRNA-RISC complex associate with GW182 protein. This association indirectly brings in a deadenylase complex, further enhancing mRNA degradation. Moreover, GW182 protein engages with various regulatory complexes and attracts the poly(A) binding protein (PABP). In addition to this, multiple deadenylating complexes are recruited to the target mRNA, effectively contributing to the degradation process (Braun et al., 2011). Subsequently, the mRNA, which has undergone deadenylation, is subjected to decapping by the miRNA-RISC complex through the recruitment of the crucial catalytic decapping protein 2 (DCP2) along with its associated decapping activators. Following decapping, the mRNA is exposed to degradation by the cytoplasmic 5′-3′ exoribonuclease 1 (Xrn1p) (Braun et al., 2011; Valinezhad Orang et al., 2014; Khan et al., 2019).

### 3.4 miRNA-mediated gene expression

While the primary function attributed to miRNAs is gene silencing, multiple studies have also documented their role in gene expression (Place et al., 2008; Xiao et al., 2017). Various mechanisms of gene expression involving miRNAs, particularly gene activation, have been documented. In the context of RNA activation, miRNAs bind to the promoter region, enhancing the presence of RNA polymerase II and transcription factors. It is postulated that AGO proteins play a pivotal role in promoting RNA activation, as they have been observed to interact with RNA polymerase II and TWIST1, a transcription factor, thereby stimulating the transcription of specific genes (Chaluvally-Raghavan et al., 2016).

Place et al. also reported gene expression mediated by miRNA promoter binding. Their study suggested that when miR-373 binds to the complementary sequence in the target gene’s promoter region, it triggers transcription activation rather than cleavage or degradation. They propose that miR-373’s ability to induce gene expression is due to an off-target effect, possibly involving the silencing of transcriptional repressor proteins. This, in turn, leads to the upregulation of E-cadherin and CSDC2 expression (Place et al., 2008). Additionally, they conducted an experiment where miR-373 was introduced into PC3 cells. They found that miR-373 did not induce other genes with promoter sequences complementary to it, suggesting that variations in the promoter environment among different cell types might influence the number of target genes activated by miRNAs. Furthermore, the study also noted that mismatches and bulges can disrupt targeted gene silencing, subsequently leading to the indirect activation of gene transcription (Place et al., 2008).

Furthermore, research has revealed that a SCZ risk-associated miRNA, miR-137, can repress MEF2A, resulting in an augmentation of mitochondrial biogenesis (Yu et al., 2007; Channakkar et al., 2020). Moreover, the same investigation demonstrated that miR-137’s repression of specific genes preserved and augmented the presence of pluripotency genes such as OCT4, SOX2, and SIRT1. This, in turn, had an effect on the differentiation process into neuronal lineage (Channakkar et al., 2020).

Nonetheless, these studies underscore that the precise mechanism behind gene upregulation remains a subject of ongoing research. A more comprehensive understanding is needed to identify the specific target molecule and the essential molecular components responsible for gene activation (Place et al., 2008; Chaluvally-Raghavan et al., 2016).

As previously mentioned, it has been established that SNPs in miRNAs or their target sites, referred to as miR-SNPs, can influence the regulatory function of miRNAs. These miR-SNPs are gaining recognition for their potential involvement in the pathological disruption of gene expression (Yu et al., 2007).

## 4 miR-SNPs gene regulation

The link between the initiation of disease pathogenesis and the existence of SNPs in miRNAs or their target sites, commonly denoted as miR-SNPs, was first established in 2007 using bioinformatics analysis (Yu et al., 2007). A single SNP in miRNAs can affect the expression of multiple genes even if the change in expression of each gene is not substantial (Sun and Zhang, 2014). As mentioned earlier SNPs in the 3′UTR region can either disrupt, weaken, or completely change the target site or change the seed region of miRNA. The average length of 3′-UTRs in the human genome is around 950 nucleotides while for highly expressed neuronal genes, the 3′ UTR extents to 1300 nucleotides (Sood et al., 2006) yet the effective miRNA-binding region is just 7-8 nucleotides. As a result, the 3′-UTR of one mRNA might include target regions for one miRNA as well as target sequences for several different miRNAs (Sood et al., 2006). This is why a single SNP in the 3′UTR has the potential to disrupt gene expression and, in turn, elevate the risk of disease onset. SNPs can also influence the genes responsible for miRNA biogenesis, such as *DROSHA* or *DICER*. This, in turn, could affect the production of mature miRNAs and subsequently alter their regulatory functions, leading to aberrations in gene expression (Haas et al., 2012; Zhou et al., 2013). In a study conducted by Zhou and colleagues, the investigation focused on analyzing SNPs within the *DICER* and *DROSHA* genes. They reported that both DICER and DROSHA are pivotal constituents of the miRNA machinery complex and play crucial roles in miRNA maturation (Zhou et al., 2013).

The SNP rs3742330, situated within the 3′untranslated region of the *DICER* gene, has been linked to an increased risk of SCZ. Changes in this region could potentially influence the stability of its mRNA and thereby affect the protein levels of the DICER. The study found a significant association between the AA genotype of rs3742330 and an increased risk of developing SCZ, suggesting that variations in this SNP might contribute to disease development, possibly through alterations in mature miRNA abundance or function (Zhou et al., 2013). Moreover, in the same investigation, a SNP known as rs10719 was identified within the 3′UTR of the DROSHA gene. However, the study did not specify the precise impact of this SNP on DROSHA’s function or its role in the processing of mature miRNAs concerning SCZ. Nevertheless, the study did observe a significant association between an SNP in DGCR8, a component of the microprocessor complex, and an elevated risk of SCZ (Zhou et al., 2013). In the case-control study carried out by Zhou and colleagues, the RNA-binding protein DGCR8 was found to harbor the SNP rs3757 within its 3′UTR. This region is vital for RNA stability, and SNPs within the 3′UTR can potentially influence transcript stability. Notably, rs3757 has been recognized as a predicted target of multiple miRNAs. In individuals with SCZ, changes in DGCR8 gene expression were observed, leading to an increase in primary miRNA processing (Santarelli et al., 2011).

Another study conducted by Sun et al. revealed the importance of SNPs on miRNA abundance. The researchers in this study identified two miR-SNPs associated with schizophrenia, inhibited maturation of miRNNAs in a male Caucasian cohort (*n* = 193 cases and 191 controls). SNPs in miR-502-C/G, miR-510-T/C were functionally relevant and linked to decreased miRNA processing, resulting in reduced precursor and mature miR-502/510 transcripts produced *in vitro*. The miR-502-C/G variant triggers bulge formation within the structure of the pre-miRNA, which changes the structure of the stem region. This bulge most likely impairs the DROSHA processing which consequently results in decreased amount of mature miR-502 both 3p and 5p. For the miR-510-T/C variant the study could not reveal at which stage the miR-510 biogenesis was hampered (Sun et al., 2009).

Even minor alterations in miRNA biogenesis genes have the potential to disrupt the regulation of target genes that have been linked to SCZ, consequently elevating the risk of the disorder. However, further research is essential to fully elucidate the biological mechanisms of SNPs in genes important for miRNA biogenesis and their role in disease development (Zhou et al., 2013).

Although more research into the SNP in miRNA biogenesis genes is needed, several SNPs in miRNAs have been identified to increase disease development. The initial research to investigate the link between SNPs in miRNA and SCZ analyzed 28 brain-expressed miRNAs in a Scandinavian cohort, consisting of Danish SCZ *n* = 420 and CTRL 1006, Swedish SCZ *n* = 163 and CTRL *n* = 177, and Norwegian SCZ *n* = 257 and CTRL 293. Interestingly, two miR-SNPs associated with schizophrenia were discovered in this study. These SNPs were in miR-206 (*p* = 0.0021 for the Danish sample) and miR-198 (*p* = 0.038 for the Norwegian sample), but their functional significance was not determined (Hansen et al., 2007). A study within the Han Chinese population showed a significant association between the ss178077483 variant found in the pre-mir-30e and SCZ risk (Xu et al., 2010). Moreover, two SNPs (hsa-pre-mir-146a rs2910164 G>C and hsa-mir-499 rs3746444 T>C) were examined for their susceptibility to SCZ among Han Chinese samples comprising 268 patients and 232 controls. Patients bearing CC genotype of rs3746444 had an elevated risk for experiencing hallucinations and lack of motivation; however, there was no statistically significant correlation between these two SNPs and SCZ (Zou et al., 2012). As mentioned previously several SNPs regulate gene expression by disrupting the binding site between the miRNA and mRNA. Additional evidence of alterations in miRNA affecting SCZ gene expression has been provided through the study by John et al., who have identified a significant association of MiRSNP rs7430 at the *PPP3CC* gene with SCZ in two independent samples (John et al., 2016). Another recent study by Molei et al. found different SNPs in miRNA binding site to increase the risk of SCZ. The significant SNPs were all in genes important for the dopaminergic system, namely, the SNPs rs165599 on the *COMT* gene and the rs200982455 *DRD2* (Molaei et al., 2022).

All of the above mentioned studies underline that miRNA processing and subsequent alteration in SCZ relevant gene expression could be a relevant targets to study in order to gain increased insights into disease mechanism.

### 4.1 SNPs affecting the interaction between miRNAs and mRNAs in dopaminergic, glutamatergic, and GABAergic systems increase the risk to develop SCZ

**FIGURE 2:**
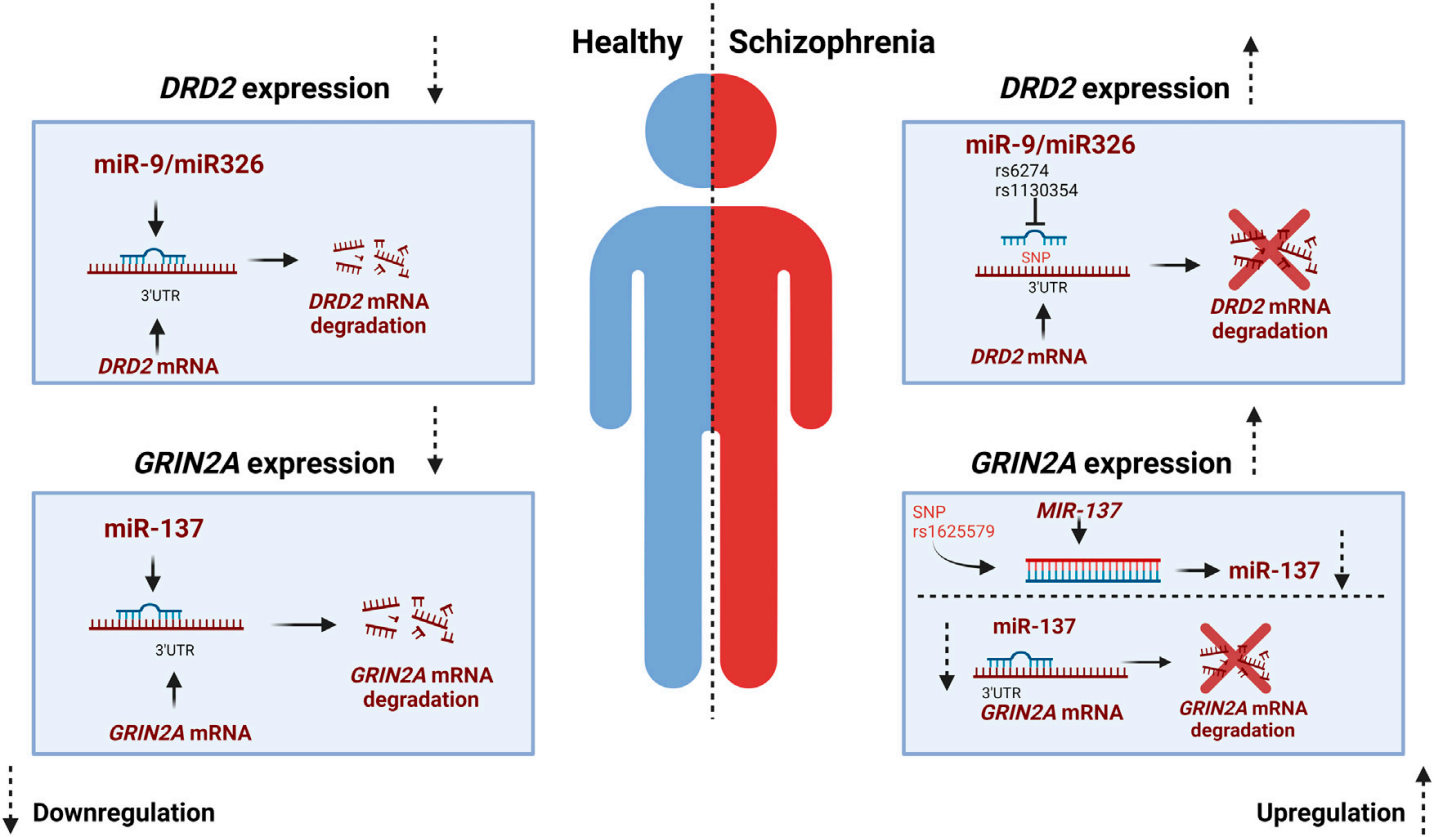
miR-SNP mediated gene expression for DRD2 and GRIN2A. In a healthy individual miR-9 and miR326 exert downregulation of *DRD2* while miR137 exert downregulation of *GRIN2A*. The miRNAs bind to the 3′UTR complementary region of the mRNA which results in degradation or repression of the gene. In individuals with Schizophrenia SNP rs6274 and rs1130354 interfere with the binding site of miRNAs and thereby an increase in gene expression of *DRD2*. While in the glutamatergic system the MIR-137 gene harbors a SNP that affects the expression of miR-137 and thereby the expression of the risk gene *GRIN2A,* which is increased in Schizophrenia. Created with BioRender.com.

As mentioned earlier, it is proposed that SCZ results from a disrupted interplay among the dopaminergic, glutamatergic, and GABAergic systems (Sigvard et al., 2023). While the study of SNPs in miRNAs and miRNA-mRNA interactions is a relatively recent area of research, SNPs in miRNA-mRNA target sites have been identified for key genes within the dopaminergic and glutamatergic system. SNPs in SCZ-associated miRNAs hold a substantial potential for contributing to SCZ risk (Shi et al., 2014). As mentioned previously DRD2, is an important presynaptic receptor of the dopaminergic system and an altered expression of this receptor has been linked to SCZ (Oda et al., 2015). MiR-9 and miR-326 have both been identified to downregulate the expression of *DRD2* gene. In contrary, SNPs located in in the seed site of miR-326 have been shown to upregulate the expression *DRD2,* consequently increasing the risk of developing SCZ (Shi et al., 2014). The 3-UTR of human *DRD2* mRNA is expected to include various miRNA binding sites including those for miR-9 and miR-326, both of which are brain-expressed miRNAs. Two well-documented SNPs, namely, rs1130354 and rs6274, are situated within the presumed targeting region shared by miR9 and miR-326. SNPs located within this target area can potentially interfere with the seed region of the miRNA, impacting its interaction with the target gene and consequently influencing target gene expression (Figure 2) (Shi et al., 2014). The rs1130354 SNP on the 3′UTR site on the *DRD2* mRNA interferes with the binding site of miR-326 which consequently upregulates the expression of *DRD2* gene. In the same research, it was observed that the SNP rs1130354 did not appear to affect miR-9, suggesting that both miRNAs regulate gene expression, albeit at distinct seed regions within the 3′UTR of the *DRD2* mRNA (Shi et al., 2014). Molaei and colleagues conducted a study that detected SNPs within the 3′UTR region of dopaminergic risk genes and in miRNAs associated with SCZ, including miR-326. However, most of these SNPs were not found to substantially elevate the risk of SCZ. In contrast, the rs200982455 SNP situated in the 3′UTR region of the *DRD2* mRNA was demonstrated to increase the risk of SCZ by a substantial factor of 3.19 (Molaei et al., 2022). When one system, in this example the dopaminergic system, is dysregulated, it impacts other systems with which it has a tight and complicated connection, namely, the glutamatergic system.

As mentioned earlier, any genetic modifications within the glutamatergic system also increase the probability of developing SCZ (Singh et al., 2022). As previously stated, *GRIN2A* plays an important role in the glutamatergic system. In addition to SNPs in *GRIN2A* that increases the risk of SCZ, SNPs in the miRNAs that regulate the expression of *GRIN2A* have also been demonstrated to raise the risk of SCZ (Wright et al., 2013). *GRIN2A* is alongside other glutamate receptor genes such as *GRIA1* and *GRIA4* a target of miR-137 (Wright et al., 2013). The potential role of miR-137 in regulating these glutamate receptors offers a plausible explanation for the atypical glutamate signaling observed in SCZ and the deficits in long-term potentiation associated with this condition. These factors could, in turn, contribute to the cognitive impairments often seen in SCZ (Wright et al., 2013). Few SNPs have been identified for miR-137 however, the SNP rs1625579 in the *MIR-137* gene is the most studied (Guella et al., 2013; Chen et al., 2018; Zhang et al., 2018; Pergola et al., 2020). Rs1625579 has been shown to be linked to lower miR-137 expression in the prefrontal cortex. This reduced miR-137 has been further associated to reduced working memory-related prefrontal activity as well as reduced activity and connectivity of the emotion processing brain network (Guella et al., 2013; Chen et al., 2018; Zhang et al., 2018; Pergola et al., 2020). In the study by Pergola et al., low expression of miR-137 and an increased target gene expression was found in the prefrontal cortex of SCZ individuals. They postulate that the SNP rs1625579 might cause the low expression of miR-137 and thereby cause the increased expression of the SCZ risk genes *GRIN2A* (Pergola et al., 2020) (Figure 2).

In a substantial GWAS conducted on a primarily European ancestry population, a potential mechanism was identified through which the SNP rs1625579 regulates the expression of the MIR-137 gene (Warburton et al., 2016). According to the study, it is hypothesized that a GWAS SNP called rs1625579 and its proximal promoter SNP rs2660304 may play a role in disease susceptibility by directly influencing the regulation of the internal miR-137 promoter (Warburton et al., 2016). miR-137 has further been identified to regulate genes important for the GABA system. *GABRA1* is responsible for encoding a chloride channel activated by ligands. This channel is a constituent of the heteropentameric receptor that responds to GABA, the principal inhibitory neurotransmitter in the brain (Steudle et al., 2020). *GABRA1* has been reported to be a potential target of miR-137 and downregulate its expression (Wright et al., 2013). GABA receptor deficiency can be expected to interfere with synaptic plasticity and result in dysregulation of the hippocampal neuron network homeostasis; hence dysregulation of *GABRA1* can result in the development of SCZ (Balan et al., 2017; Li et al., 2018b). Another inhibitory complex potentially targerted by miRNAs are genes encoding the GABAB receptors. Intriguingly, miR-466b-5p, miR-410-5p, miR-3583-3p upregulation has been linked to *GABBR2* expression, however, the study did not specify which miRNA targets *GABBR2* (Y. Li et al., 2018)*. GABBR2* encodes a multi-pass membrane protein that is a member of the G-protein coupled receptor 3 family and the GABA-B receptor subfamily. Potential disruption of the receptor complex and signal transmission is another promising avenue to unravel the disease mechanisms in SCZ. Nevertheless, studies to this date investigating the role of SNPs interfering into miRNA-mRNA interaction in genes relevant for the GABA system are sparse. Additional research is certainly required to substantiate the evidence of SNPs within miRNAs that modulate critical genes in the GABA system, especially concerning their implications for SCZ.

## 5 Conclusion

In summary, this review underscores the substantial impact of SNPs, miRNAs, and notably the emerging roles of SNPs within miRNAs in the development of SCZ. Specifically, SNPs in the dopaminergic, glutamatergic, and GABAergic systems, operating at both the mRNA and miRNA levels, have been recognized as pivotal players. These SNPs can heighten the risk of SCZ by interfering with or modifying the interaction between miRNAs and their target sites within mRNAs. The resulting dysregulation hinders healthy brain development and contributes to an elevated susceptibility to SCZ.
